# Repurposing harmaline as a novel approach to reverse *tmexCD1-toprJ1*-mediated tigecycline resistance against *klebsiella pneumoniae* infections

**DOI:** 10.1186/s12934-024-02410-4

**Published:** 2024-05-24

**Authors:** Jindian Yang#, Lei Xu#, Yonglin Zhou, Minhe Cui, Dejun Liu, Jianfeng Wang, Yang Wang, Xuming Deng

**Affiliations:** 1 State Key Laboratory for Diagnosis and Treatment of Severe Zoonotic Infectious Diseases, Key Laboratory for Zoonosis Research of the Ministry of Education, Institute of Zoonosis, and College of Veterinary Medicine, Jilin University, Changchun, 130062 China; 2 Beijing Key Laboratory of Detection Technology for Animal-Derived Food Safety, College of Veterinary Medicine, China Agricultural University, Beijing, China; 3 Key Laboratory of Ministry of Education for Conservation and Utilization of Special Biological Resources in the Western China, School of Life Sciences, Ningxia University, Yinchuan, China; 4 Jilin Province Mushuo Livestock Farming Co., Ltd., Jilin, China

**Keywords:** Harmaline, *tmexCD1-toprJ1*, Tigecycline, *Klebsiella pneumoniae*

## Abstract

**Background:**

A novel plasmid-mediated resistance–nodulation–division (RND) efflux pump gene cluster *tmexCD1-toprJ1* in *Klebsiella pneumoniae* tremendously threatens the use of convenient therapeutic options in the post-antibiotic era, including the “last-resort” antibiotic tigecycline.

**Results:**

In this work, the natural alkaloid harmaline was found to potentiate tigecycline efficacy (4- to 32-fold) against *tmexCD1-toprJ1-*positive *K. pneumoniae*, which also thwarted the evolution of tigecycline resistance. *Galleria mellonella* and mouse infection models in vivo further revealed that harmaline is a promising candidate to reverse tigecycline resistance. Inspiringly, harmaline works synergistically with tigecycline by undermining *tmexCD1-toprJ1-*mediated multidrug resistance efflux pump function via interactions with TMexCD1-TOprJ1 active residues and dissipation of the proton motive force (PMF), and triggers a vicious cycle of disrupting cell membrane integrity and metabolic homeostasis imbalance.

**Conclusion:**

These results reveal the potential of harmaline as a novel tigecycline adjuvant to combat hypervirulent *K. pneumoniae* infections.

**Supplementary Information:**

The online version contains supplementary material available at 10.1186/s12934-024-02410-4.

## Introduction

Antimicrobial resistance (AMR), resulting from the horizontal spread of resistance genes mediated by chromosomal mutations or mobile elements such as plasmids, has become one of the most remarkable public health challenges worldwide since the 1970s [1]. One Health surveillance for antimicrobial resistance has been promoted by the scientific community and by international organizations for more than a decade. To assist countries in the development of surveillance systems, the WHO Advisory Group on Integrated Surveillance of Antimicrobial Resistance released its new guidelines on the application of a One Health approach to surveillance of antimicrobial resistance in foodborne bacteria in 2017 [2]. Under these circumstances, multidrug-resistant bacteria have developed at a horrifying rate and endured, which has stressed health-care systems around the world. Tigecycline, as a semisynthetic extra gastrointestinal glycylcyclic antibiotic with broad-spectrum activity [3], has been touted as a last line agent against bacterial infections since its discovery in 1993 and introduction into the clinic in 2005 [4]. However, with the widespread use and misuse of antibiotics, resistance to tetracycline antibiotics has emerged globally, with dangerous implications for public health and food safety [5].

However, recent studies have identified *tmexCD1-toprJ1*, a novel multidrug resistance efflux pump gene cluster carried by the plasmid of *K. pneumoniae* that mediates high levels of resistance to drugs such as tigecycline [6], further detailing the antimicrobial susceptibility and genetic and functional characterization of *tmexCD1-toprJ1*-positive clinical strains, as well as potential interspecies transmission pathways of *tmexCD1-toprJ1* gene clusters [7]. Epidemiologic investigations identified plasmid-encoded variants of transmissible RND efflux pump genes, such as *tmexCD2-toprJ2* from diseased patients in China [8] and *tmexCD3-toprJ3* in *Klebsiella* spp. from municipal sludge in Vietnam [9], suggesting that plasmid-mediated mechanisms for RND efflux are emerging and spreading on a large scale and on multiple scales around the globe. Alarmingly, recent findings revealed that the *tmexCD1-toprJ1* and *tet*(X) genes were simultaneously detected in high abundance in animal fecal samples, which is seriously threatening the efficacy of clinical therapeutic options for multidrug-resistant pathogens [10].

Exhibiting a highly diverse genetic context and potential for transmission between hosts, *tmexCD-toprJ* has been metastasized in a wide range of clinical pathogens. Hence, there is an urgent need to monitor and control the spread of the *tmexCD1-toprJ1* gene cluster and explore novel RND-type efflux pump TMexCD1-TOprJ1 inhibitors, which would be potent pharmacologically active ingredients and anti-infective therapeutic strategies for the control of drug-resistant *tmexCD1-toprJ1*-producing *K. pneumoniae* infections.

Accumulating evidence has demonstrated that antibiotic adjuvant strategies hold a promise in extending the lifespan of existing antibiotics [11]. Harmaline, a β-carboline alkaloid, has anti-inflammatory [12], antiviral [13], and anticancer activities [14]. However, the role and mechanism of harmaline as a tigecycline co-adjuvant against *tmexCD1-toprJ1*-positive *K. pneumoniae* have not been fully explored. Therefore, this study focuses on tigecycline resistance mediated by the efflux pump gene cluster *tmexCD1-toprJ1* to address the clinically relevant problem of drug resistance in *K. pneumoniae*. The study demonstrated the potential of harmaline as synergistic adjuvant of tigecycline to combat *tmexCD1-toprJ1*-positive pathogens infections.

## Materials and methods

### Reagents

Harmaline was purchased from Derick Biotechnology Co., Ltd. (Chengdu, China), and the purity of which was confirmed to be > 95% by high-performance liquid chromatography (HPLC) analysis. Tigecycline with an HPLC purity > 98% was supplied by Shanghai Yuanye Bio-Technology Co., Ltd.

### Bacterial strains and culture conditions

The bacterial strains used in this study were listed in Supplementary Table 1. The bacterial strains were cultured in Luria–Bertani (LB) broth or Mueller-Hinton broth (Qingdao Haibo Biotechnology Co., Ltd.) at 37 °C with shaking at 180 rpm.

### Animals and cell culture

*Galleria mellonella* were purchased from Huiyude Biotechnology Co., Ltd. (Tianjin, China), and Balb/C and KM mice were purchased from Changsheng Biotechnology Co., Ltd. (Liaoning, China). Mice were kept in strict accordance with the regulations for the Administration of Laboratory Animals (11-14-1988) approved by the State Council of the People’s Republic of China. All animal experiments were approved by the Animal Care Committee and the Welfare and Research Ethics Committee of Jilin University (Approval No. SY202309036) and were conducted in accordance with the relevant guidelines and regulations of the committees. Caco-2 and J774A.1 cells (laboratory original) were cultured in DMEM + 10% FBS + 1% P/S at 37 °C.

### Antimicrobial susceptibility testing

The minimum inhibitory concentration (MIC) of all drugs was determined using the standard microdilution method according to CLSI Guidelines 2018 [15]. Briefly, the compounds were diluted twofold in a 96-well microtiter plate containing 100 µL of Mueller-Hinton broth, followed by the addition of the prepared overnight bacterial culture (5 × 10^5^ CFU/mL). After 20–24 h of incubation at 37 °C, the MIC was defined as the lowest drug concentration for which no visible bacterial growth was observed.

The synergistic activity of the compounds and antibiotics was determined by the modified checkerboard method. The fractional inhibitory concentration (FIC) index values were calculated with the following equation: FICI = (MIC of the compound in combination/MIC of the compound) + (MIC of the antibiotic in combination/MIC of the antibiotic). The antimicrobial combinations were defined as synergistic if FICI ≤ 0.5 [16].

### Growth curves

The overnight cultures were inoculated in fresh Mueller-Hinton broth until the logarithmic growth stage (OD_600nm_ = 0.3). Then, the indicated concentrations of harmaline (0, 64, or 128 mg/L) were added to the bacterial cultures, which were then incubated at 37 °C with shaking at 180 rpm. The OD_600nm_ values were measured spectrophotometrically at 30-min intervals.

### Time-dependent killing

Overnight bacterial cultures (5 × 10^5^ CFU/mL) were incubated with harmaline (128 mg/L), tigecycline (4 mg/L) or the combination. At the indicated time points (0, 1, 3, 7, 10, 12, and 24 h), the different treatment samples were collected and plated for colony counting. An increase in lethality of ≥ 2log_10_ CFU/mL with the combination compared to the most active single agent is considered synergistic. Antagonism is defined as a decrease in kill of ≥ 2log_10_ CFU/mL with the combination compared to the most active single drug. There is no indifference when the killing effect of the two drugs in combination differs within 2log_10_ CFU/mL from the killing effect of either drug alone.

### Post antibiotic effect (PAE) assay

*K. pneumoniae* T2 was cultured at 37 °C in Mueller-Hinton broth to the log-phase. Then, the diluted bacteria (1 × 10^8^ CFU/mL) were exposed to harmaline (0, 64, or 128 mg/L), tigecycline (0, 4, or 8 mg/L) or the conbinations for 2 h at 37 °C. After incubation, samples were removed by diluting 1:1000 with Mueller-Hinton broth. Viable counts were determined immediately after dilution and at 2 h intervalor at least 10 h. PAE determination was calculated as PAE (h) = T-C, where T is the time (h) that required to increase by tenfold for the drug-treated viable count from the moment of dilution and C is the time (h) required to increase by tenfold for cell density without treatment.

### Combination disc test

Bacterial cultures were diluted with Mueller-Hinton broth to OD_600nm_ values of 0.1 and 0.3 and were further plated on solid media containing 0, 32, 64, or 128 mg/L harmaline. Then, the 15 µg tigecycline discs were gently placed on the agar plate. The size of inhibition zones were observed and recorded the next day.

### Resistance evolution analysis

The effect of harmaline on the development of acquired resistance was determined using serially passaged cultures [17]. Briefly, *K. pneumoniae* T2 cultures were incubated with tigecycline (16 mg/L) or harmaline in combination with tigecycline (128 + 16 mg/L) at 37 °C. The tigecycline MICs against the tested bacteria were measured for 30 days continuously.

### Mutant prevention concentration (MPC) determination

Bacteria (*K. pneumoniae* T2) at 10^10^ CFU/mL were placed on LB-agar plates containing tigecycline alone (0, 4, 8, 16, 32, 64, 128, 256, 512, 1024, or 2048 mg/L) or in combination with harmaline at different concentrations (0, 32, 64, 128, or 256 mg/L). The plates were then cultured at 37 °C for 72 h, and the lowest tigecycline concentration that limited bacterial growth at each set of concentration harmaline treatment was defined as the MPC [18].

### Determination of efflux pump function

The fluorescent dye Rhodamine B (Sigma, Germany) was employed to assess the effect of harmaline on efflux pump function. *K. pneumoniae* T2 in the logarithmic growth stage (OD_600nm_ = 0.6) was washed and incubated with Rhodamine B (10 µM) at 37 °C and 180 rpm for 30 min. The cultures were then treated with different concentrations of harmaline (0, 32, 64, or 128 mg/L), tigecycline (0, 4, or 8 mg/L), and the combinations for 1 h at 37 °C. Finally, the fluorescence intensity of the bacterial supernatant (8, 000 rpm, 10 min) was measured with an excitation wavelength of 553 nm and an emission wavelength of 627 nm.

### Ethidium bromide (EtBr) efflux assay

To assess the inhibitory effect of harmaline on the multidrug efflux pump, we performed an ethidium bromide (EtBr) efflux assay based on previous studies [19]. Bacteria in the logarithmic growth phase (OD_600nm_ = 0.6) were collected, washed and incubated with different concentrations of harmaline (0, 32, 64, or 128 mg/L), tigecycline (0, 4, or 8 mg/L), and the combinations for 1 h at 37 °C. Subsequently, 10 µM EtBr (Sigma, Germany) was supplemented, and the samples were incubated for 1 h at 37 °C; the efflux pump inhibitor carbonyl cyanide-3-chloro phenylhydrazone (CCCP) (100 µM, Sigma, Germany) served as a positive control. After being centrifuged at 8, 000 rpm for 10 min, the precipitates were collected, washed and resuspended in PBS. The efflux efficacy of EtBr was monitored using an excitation wavelength of 530 nm and an emission wavelength of 600 nm.

### Inner membrane permeability

Bacteria cultures in logarithmic growth were collected, washed and resuspended to an OD_600nm_ of 0.6, the cultures were then treated with different concentrations of harmaline (0, 64, or 128 mg/L), tigecycline (0, 4, or 8 mg/L) or the combinations for 1 h. Prodium iodide (PI) (Yuanye, China) was added to a final concentration of 10 nM and the samples were incubated for 30 min in the dark. The fluorescence intensity was measured at an excitation wavelength of 535 nm and an emission wavelength of 615 nm.

### Determination of outer membrane permeability

The fluorescent probe N-phenyl-l-naphthylamine (NPN) (Macklin, China) was employed to assess the effect of harmaline on outer membrane permeability. Briefly, bacterial cultures (OD_600nm_ = 0.5) were pretreated with various concentrations of harmaline (0, 64, or 128 mg/L), tigecycline (0, 4, or 8 mg/L), or the combinations at 37 °C for 1 h. The cells were incubated with NPN (10 µM) and absorbance was measured with an excitation wavelength of 350 nm and an emission wavelength of 420 nm.

### Extracellular β-galactosidase measurement

Bacterial cultures (OD_600nm_ = 0.6) were treated with different concentrations of harmaline (0, 64, or 128 mg/L), tigecycline (0, 4, or 8 mg/L) or the combinations at 37 °C for 1 h. A final concentration of 10 mM 2-nitrophenyl-β-D-galactopyranoside (ONPG) (Macklin, China) was then added and samples were incubated for an additional 30 min; then, the absorbance at 420 nm was recorded.

### Membrane fluidity

Overnight bacterial cultures (OD_600nm_ = 0.7) in the presence or absence of harmaline (0, 64, or 128 mg/L), tigecycline (0, 4, or 8 mg/L), or the combinations were incubated with 40 µM 8-anilino-1-naphthalenesulfonic acid (ANS) (Macklin, China) for 30 min. The fluorescence intensity was determined at excitation and emission wavelengths of 385/473 nm.

### Membrane depolarization analysis

Bacteria in the logarithmic phase (OD_600nm_ = 0.6) were collected, washed, and incubated with 10 µM 3,3’-dipropylthiadicarbocyanine iodide (DiSC3(5)) (Sigma, Germany). The fluorescence intensity was measured continuously with an excitation wavelength of 622 nm and an emission wavelength of 670 nm for 8 min. Then, the corresponding concentrations of harmaline (0, 32, 64, or 128 mg/L), tigecycline (0, 8 mg/L), and the combinations (64 + 8, 128 + 8 mg/L) were added and the fluorescence was measured for an additional 32 min (two-minute interval).

### Proton gradient measurement

*K. pneumoniae* T2 (OD_600nm_ = 0.6) was incubated with 10 µM 2’,7’-Bis(2-carboxyethyl)-5(6)-carboxyfluorescein acetoxymet (BCECF-AM) (Merck, Germany) at 37 °C for 30 min and treated with the indicated concentrations of harmaline (0, 32, 64, or 128 mg/L), tigecycline (0, 8 mg/L), or the combinations (64 + 8, 128 + 8 mg/L) for an additional 30 min. The fluorescence intensity was determined at excitation and emission wavelengths of 488/525 nm. The fluorescence at the beginning was subtracted by the fluorescence at the 40 min.

### Estimation of zeta potential

Bacterial cultures (OD_600nm_ = 0.5) were treated with different concentrations of harmaline (0, 64, or 128 mg/L), tigecycline (0, 4, or 8 mg/L), or the combinations at 37 °C for 2 h. The Zeta potential was measured by dynamic light scattering at 25 °C using a Nano Zeta sizer (ZS) ZEN3600.

### Molecular docking

Using AutoDock Vina software (http://vina.scripps.edu/), the ligands and proteins needed for molecular docking were prepared, and for the target proteins, the crystal structures were obtained from the PDB database (https://www.rcsb.org/); the crystal structures required preprocessing, including the removal of hydrogenation, the modification of amino acids, the optimization of energies, and the tuning of force field parameters. For the target protein, the ligand structure was downloaded from the PDB database (https://pubchem.ncbi.nlm.nih.gov/), after which the low-energy conformation of the ligand structure was satisfied. Finally, this target structure was molecularly docked with the active ingredient structure utilizing vina in pyrx software (https://pyrx.sourceforge.io/). This complex was visualized using PyMOL (https://pymol.org/2/), and the 2D plots were visualized and analyzed using Discovery Studio 2020 Client (https://discover.3ds.com/discovery-studio-visualizer- download).

### Amino acid site-specific mutation

The *tmexC1*, *tmexD1*, and *toprJ1* genes were amplified by using primers with the genomic DNA of *K. pneumoniae* T2 as the template. The product was digested with BamHI and XhoI and cloned into similarly digested pET28a/pGEX-6P-1 to generate pET28a-*tmexC1*/*toprJ1* and pGEX-6P-1-*tmexD1*. The recombinant plasmids were used as templates and the list of primers used for mutagenesis is shown in Supplementary Table 2. The PCR products were added to Dpn I enzyme to digest the methylation template and then transformed into *E. coli* DH5α/BL21(DE3) strain to obtain the recombinant strains. The clone was confirmed by sequencing.

### Protein expression and purification

Recombinant pET28a-TMexC1/TOprJ1 were purified by HisPur™ Ni-NTA affinity chromatography. TMexD1 was expressed by fusion with glutathione-S-transferase (GST) in the *E. coli* BL21(DE3) strain and gene-specific primers were shown in Supplementary Table 3. The cultures were induced with isopropyl-β-d-thiogalactoside (IPTG) overnight at 16 °C, centrifuged at 7, 000 rpm for 30 min, and then lysed for purification. The purity of the proteins was determined by separation on SDS‒PAGE gels, and the protein concentrations were determined by a Quawell DNA/Proteins Analyzer.

### Circular dichroism (CD) spectra analysis

The secondary structures of the TMexC1/TMexD1/TOprJ1 proteins were further analyzed by circular dichroism (CD). Briefly, TMexC1, TMexD1, and TOprJ1 proteins in the presence or absence of harmaline (128 mg/L) were scanned in the range of 190 to 260 nm using a CD spectrophotometer (MOS-500; Bio-Logic) according to the methods of our previous study [20].

### Reverse transcriptase PCR (RT‒PCR) analysis

The effect of harmaline on the transcription of *tmexC1*, *tmexD1*, and *toprJ1* was investigated using RT‒PCR analysis. Briefly, overnight *K. pneumoniae* T2 was incubated with concomitant addition of harmaline at final concentrations of 0, 64, or 128 mg/L for 4 h. Total RNA was then extracted, and pretreated. RNA was reverse transcribed to cDNA using the NovoScript® Plus All-in-one 1st Strand cDNA Synthesizer (Mei 5, China) and gene-specific primers were shown in Supplementary Table 4. The transcriptional levels of *tmexC1*, *tmexD1*, and *toprJ1* in each sample were determined by quantitative real-time analysis using the 2^−ΔΔCt^ method. In addition, the 16 S ribosomal RNA gene served as an internal control.

### Western blotting analysis

*K. pneumoniae* T2 (OD_600nm_ = 0.3) was treated with the indicated concentrations of harmaline (0, 32, 64, or 128 mg/L) and incubated for 4 h. Then, the bacterial cultures were centrifuged and collected (12, 000 rpm, 10 min). Western blotting assays were employed to analyze the effect of harmaline on TMexC1/TMexD1/TOprJ1 expression. In brief, the protein was separated by 10% SDS‒PAGE and transferred to polyvinylidene difluoride (PVDF) membranes. After blocking for 2 h, the membranes were incubated with the specific primary antibodies and then with HRP-conjugated goat anti-mouse or anti-rabbit secondary antibody. The membranes were visualized using an enhanced chemiluminescence ECL kit (Biosharp, China).

### Intracellular ATP determination

Bacteria in the logarithmic growth phase (OD_600nm_ = 0.6) were washed and incubated with different concentrations of harmaline (0, 64, or 128 mg/L), tigecycline (0, 4, or 8 mg/L), or the combinations at 37 °C for 2 h. The intracellular adenosine triphosphate (ATP) level was determined using the Enhanced ATP Assay Kit (Beyotime, China) according to the manufacturer’s instructions.

### ROS level determination

The level of reactive oxygen species (ROS) was measured with 2′, 7′-dichlorodihydrofluorescein diacetate (DCFH-DA) (Beyotime, China) according to the manufacturer’s instructions. Briefly, logarithmically growing bacteria (OD_600nm_ = 0.6) in the presence or absence of harmaline (0, 64, or 128 mg/L), tigecycline (0, 4, or 8 mg/L), or the combinations were incubated with DCFH-DA (10 µM) for 30 min at 37 °C. The fluorescence intensity was measured at an excitation wavelength of 488 nm and an emission wavelength of 525 nm.

### NAD^+^/NADH level determination

Bacteria in the logarithmic growth phase (OD_600nm_ = 0.6) were washed and treated with different concentrations of harmaline (0, 64, or 128 mg/L), tigecycline (0, 4, or 8 mg/L), or the combinations for 2 h at 37 °C. According to the manufacturer’s instructions, the NAD^+^/NADH level was determined at 450 nm.

### SOD and GSH activity determination

The bacterial cultures in the logarithmic growth phase (OD_600nm_ = 0.6) were collected and incubated with the indicated concentrations of harmaline (0, 64, or 128 mg/L), tigecycline (0, 4, or 8 mg/L), or the combinations for 2 h at 37 °C. Then, superoxide dismutase (SOD) and glutathione (GSH) activity were measured using Beyotime (China) under the manufacturer’s instructions.

### Cytotoxicity evaluation

The cytotoxicity of harmaline/tigecycline and harmaline in conbinations of tigecycline against J774A.1 and Caco-2 cells was determined using a Cytotoxicity Detection Kit (Roche, Switzerland). Cells were treated with or without harmaline (0, 4, 8, 16, 32, 64, or 128 mg/L), or co-incubated with harmaline (0, 64, or 128 mg/L), tigecycline (0, 4, or 8 mg/L), or their combinations for 5 h, and the cytotoxicity in the cultured supernatants was measured at OD_492nm_. Cells treated with 0.2% Triton X-100 were employed as a positive control, and untreated cells were used as a negative control.

### Animals

#### *Galleria mellonella* infection model

*Galleria mellonella* were purchased from Huiyude Biotech Company (Tianjin, China) and infected with *K. pneumoniae* T2 at a dose of 10^6^ CFUs (10 µL) for survival experiments. Then, the *Galleria mellonella* were randomly divided into the following 4 groups (*n* = 10 per group): the infection group, tigecycline monotherapy (1 mg/kg) group, harmaline monotherapy (1 mg/kg) group, and combination (1 + 1 mg/kg) group. The survival of *Galleria mellonella* was monitored for 20 h post infection for survival analysis.

*Galleria mellonella* used for *K. pneumoniae* colonization analysis were divided into treatment groups (*n* = 8). Bacteria (2 × 10^5^ CFUs, 10 µL) were administered at 0, 24, and 48 h. Tigecycline (1 mg/kg), harmaline (1 mg/kg), and tigecycline + harmaline (1 + 1 mg/kg) were administered at 0, 12, 24, 36, and 48 h post infection. At 60 h post infection, the infected *Galleria mellonella* were homogenized, diluted and plated for bacterial counting.

## In vivo toxicity evaluation

After administration of 200 mg/kg harmaline each to 6- to 8-week-old female KM mice (*n* = 3) and 6- to 8-week-old male KM mice (*n* = 3), the status and body weights of mice were monitored daily for 14 days.

## Mouse infection assays

For survival analysis, the mice were infected with a dose of 1.6 × 10^10^ CFUs *K. pneumoniae* T2 (30 µL) via nasal drip. Then, the mice were randomly divided into the following 4 groups (*n* = 10 per group): the infection group, tigecycline monotherapy (10 mg/kg) group, harmaline monotherapy (20 mg/kg) group, and tigecycline + harmaline (10 + 20 mg/kg) treatment group. Harmaline was administered by gavage, and tigecycline was administered by subcutaneous injection at 12-hour intervals. The survival of the mice was monitored continuously for 7 days.

Additionally, the mice were nasally infected at a dose of 10^7^ CFUs (30 µL) at 0, 24, and 48 h post infection for bacterial burden experiments and then randomly divided to 4 groups (*n* = 6 per group); tigecycline monotherapy (10 mg/kg), harmaline monotherapy (20 mg/kg), and tigecycline + harmaline (10 + 20 mg/kg) were administered every 12 h. Harmaline and tigecycline were administered by gavage and subcutaneous injection, respectively. Mice were euthanized at 60 h post infection, and the lungs were harvested for microbial plating. In addition, inflammatory cytokines (TNF-α, IL-1β, and IL-10) levels in livers were determined using ELISA kits (BioLegend, the United States) following the manufacturer’s instructions.

### Data analysis

All data are expressed as the mean ± SD from biological replicates. Statistical significance was determined using GraphPad Prism 9.5.1 software via one-way ANOVA or the log-rank (Mantel‒Cox) test, and comparisons among more than two groups were assessed by two-way ANOVA, which is shown with ns (no significance) (*p* ≥ 0.05), *p < 0.05, **p < 0.01, *** *p* < 0.001, and **** *p* < 0.0001.

## Results

### Harmaline, as a potent tigecycline adjuvant, works synergistically with tigecycline against *tmexCD1-toprJ1*-positive *K. pneumoniae*

**Fig. 1:**
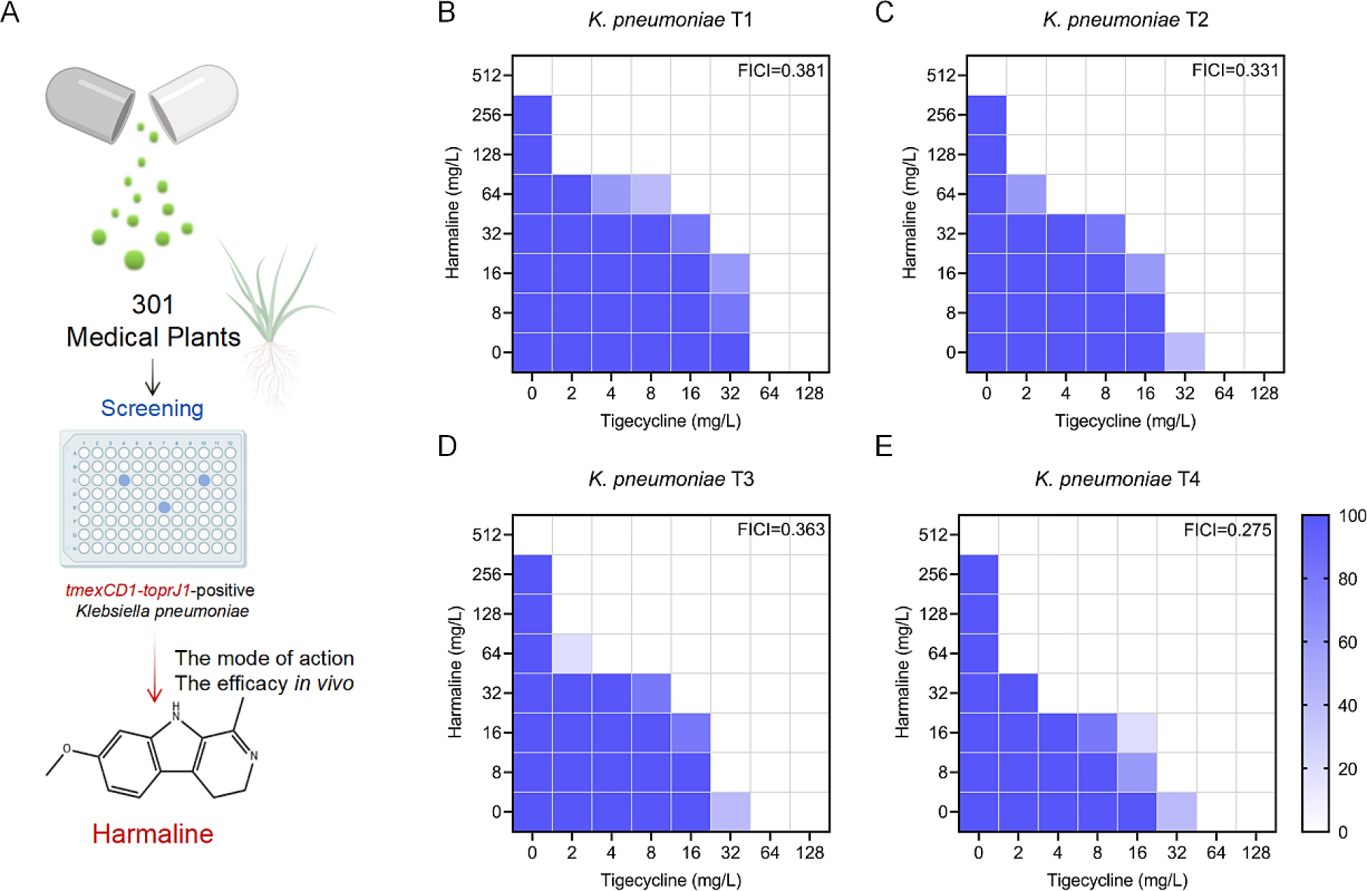
Harmaline significantly enhanced tigecycline activity against *tmexCD1-toprJ1-*positive bacteria. **A** The protocol for screening antimicrobial compounds in this study. **B-E** FIC index of the combination of harmaline and tigecycline against four strains of *tmexCD1-toprJ1*-positive bacteria. The darker the blue color, the higher the probability of long bacteria. An FIC index ≤ 0.5 indicates synergy. Data represent five biological replicates

Medicinal plants and plant extracts serve as a natural drug repository, offering effective treatments against pathogenic bacteria and playing a crucial role in the discovery of novel antibiotics [21–23]. Our existing library of plant extracts (301 species) was employed to screen for *tmexCD1-toprJ1-*mediated efflux pump inhibitors via an antimicrobial susceptibility assay (Fig. 1A). Remarkably, harmaline showed potential synergistic activity by restoring the susceptibility of drug-resistant bacteria harboring *tmexCD1-toprJ1* to tigecycline, as evidenced by a fractional inhibitory concentration index (FICI) of less than 0.5 (Fig. 1B through E). To investigate whether this synergistic effect was specific to tigecycline, we evaluated harmaline synergistic effects with other tetracycline antibiotics (methacycline, doxycycline, and tetracycline) using the same method. As shown in Figure S1A, the FIC index for the combination of harmaline and other tetracycline antibiotics were all greater than 0.5, demonstrating no synergy [24]. Additionally, we tested non-*tmexCD1-toprJ1* carrying strains (*E. coli* J53p47EC (*tet* (X4)), *K. pneumoniae* 12016p47EC (*tet* (X4)), *E. coli* 47R (*tet* (X4)), *A. baumannii* 34AB (*tet* (X3))) and no synergistic effects were found between harmaline and tigecycline, as their FIC were all greater than 0.5 (Figure S1B). To investigate the broad-spectrum synergistic potential of harmaline, we sought to explore the potency of the combination of harmaline with diverse antibiotics. As depicted in Figure S1C, harmaline potentiates the activity of multiple antibiotics, including polymyxin B and ceftiofur sodium [24], suggesting that harmaline is a potential broad-spectrum antibiotic adjuvant for combating *tmexCD1-toprJ1-*positive *K. pneumoniae*.

**Fig. 2:**
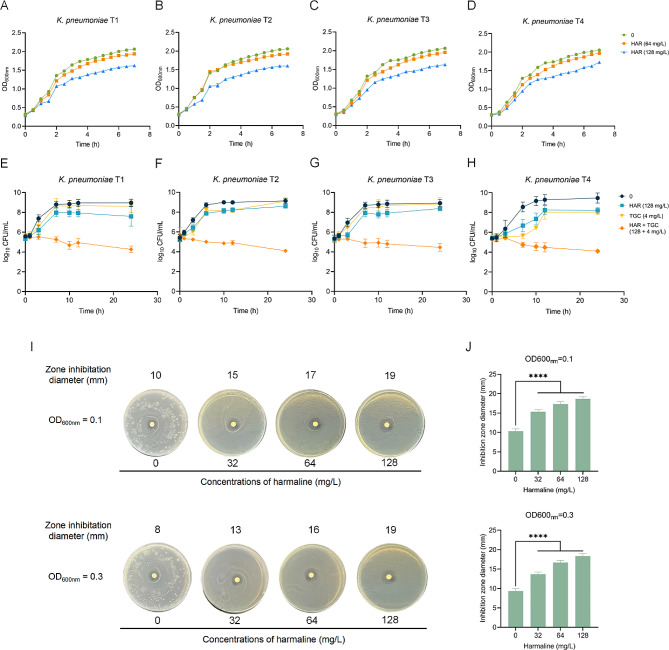
Synergistic bactericidal effects of harmaline and tigecycline *in vitro.***A-D** Growth curves of *tmexCD1-toprJ1*-positive isolates with the presence or absence of harmaline. **E-H** Time-killing curves of harmaline/tigecycline alone or in combination against *tmexCD1-toprJ1*-positive bacteria. **I-J** Determination of the diameter of the zone of inhibition of *K. pneumoniae* T2 by harmaline versus tigecycline (15 µg) (****, *p* < 0.0001). Data represent three biological replicates

In order to verify harmaline alone does not directly kill bacteria, growth curves were performed under different concentrations of harmaline treatment. Harmaline administration showed negligible effects on bacterial growth at the effective concentrations (Fig. 2A through D). We further probed the synergistic effects of harmaline and tigecycline in vitro, as depicted in Fig. 2E through H, and the combination of harmaline plus tigecycline exhibited potent synergistic effects against *tmexCD1-toprJ1-*positive *K. pneumoniae.* As shown in Figure S2, synergistic treatment of *K. pneumoniae T2* with harmaline in combination of tigecycline resulted in a visible increase in PAE. The results indicated that harmaline in combination of tigecycline prolonged effects on bacterial growth. Additionally, the combined disk tests also showed the synergistic potential between harmaline and tigecycline, as evidenced by the significantly larger zone of inhibition (Fig. 2I and J).

### Harmaline prevents the evolution of tigecycline resistance

**Fig. 3:**
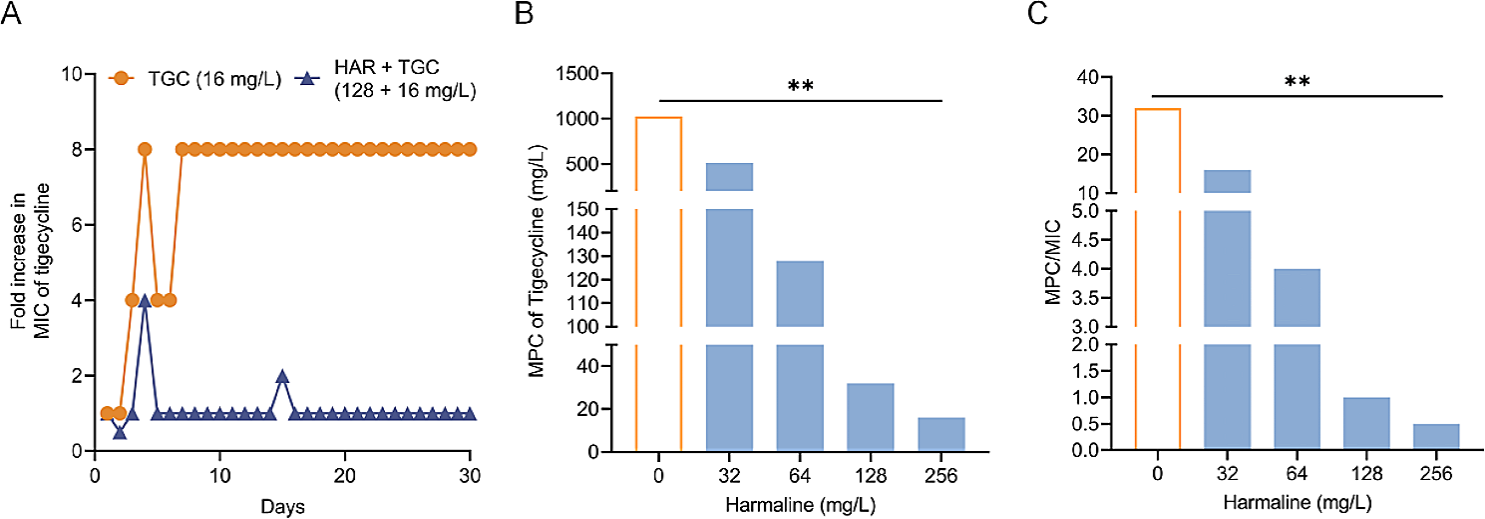
Harmaline blocks the evolution of tigecycline resistance in *tmexCD1-toprJ1*-positive bacteria. **A** Changes in tigecycline MIC values against *K. pneumoniae* T2 in the presence of tigecycline (16 mg/L) or a combination of 128 mg/L harmaline and 16 mg/L tigecycline within 30 days of passaging. **B** Mutation prevention concentration (MPC) of *K. pneumoniae* T2 against tigecycline in the presence of harmaline at concentrations of 0, 32, 64, 128, or 256 mg/L. **C** Ratio of MPC to MIC (**, *p* < 0.01). Three biological replicates per dataset were included

Preventing the evolution of bacterial resistance is of the utmost importance [25]. To investigate the potential of harmaline in preventing the evolution of tigecycline resistance, serial passages of *tmexCD1-toprJ1-*positive *K. pneumoniae* supplemented with tigecycline in the presence or absence of harmaline for 30 consecutive days were performed. Consequently, the evolution of tigecycline resistance was observably suppressed in the presence of harmaline (Fig. 3A). MPC is an important indicator of resistance changes [26]. Furthermore, we determined the mutant prevention concentration (MPC) of *K. pneumoniae* T2 against tigecycline under different harmaline concentrations. As the harmaline concentration increased, both the MPC and the ratio of MPC/MIC significantly decreased, indicating a narrower mutation selection window in the presence of harmaline (Fig. 3B and C). These findings suggest that the combined use of harmaline and tigecycline effectively minimized the *de novo* emergence of tigecycline in *tmexCD1-toprJ1*-positive bacteria.

### Harmaline inhibits efflux pump function by dissipating the proton motive force

**Fig. 4:**
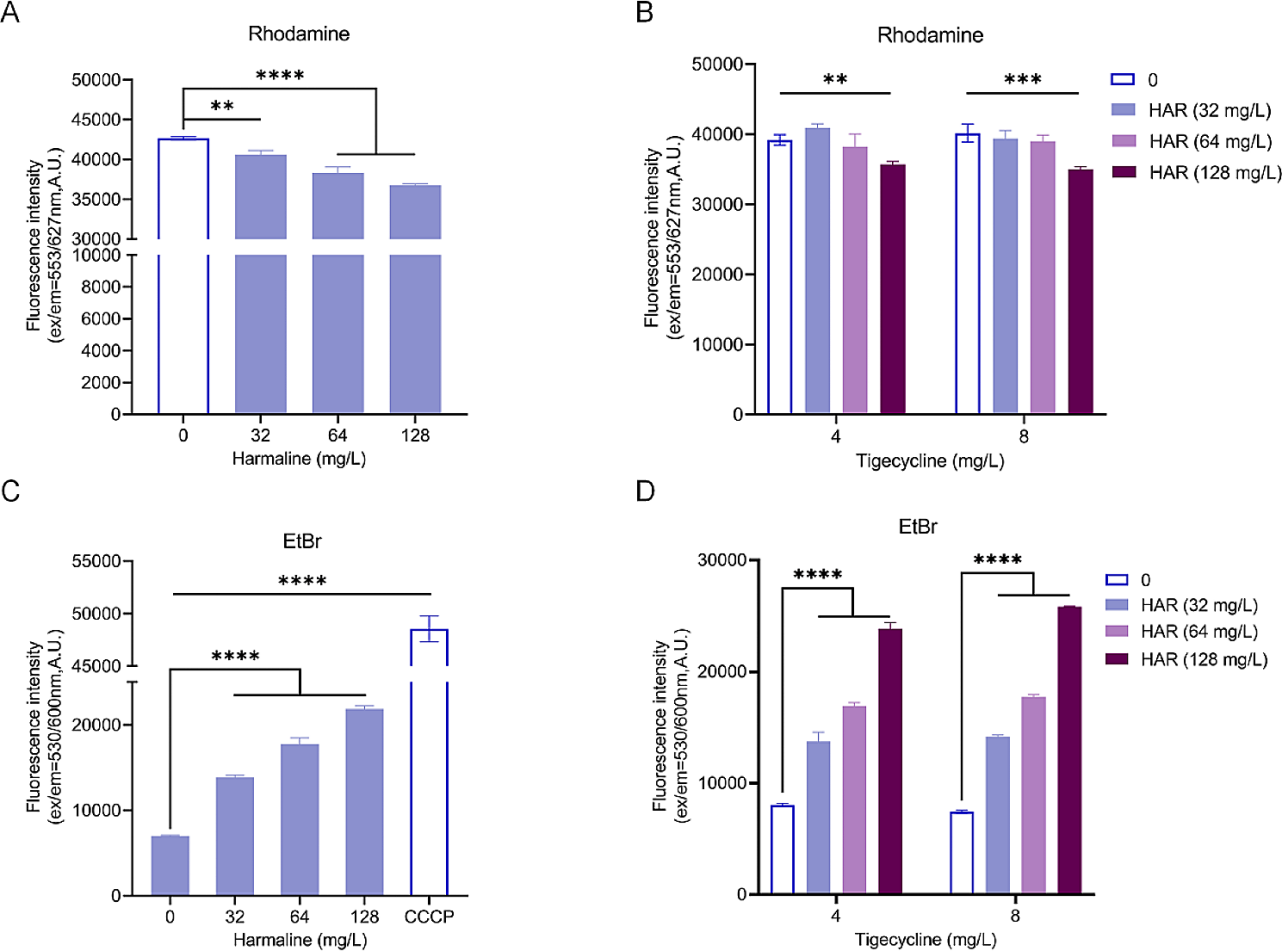
Harmaline inhibits the function of efflux pumps. The fluorescence intensity of Rhodamine B **A-B** and Ethidium bromide (EtBr) **C-D** with the indicated concentrations of harmaline alone or combined with tigecycline was monitored to characterize the efflux pump function (**, *p* < 0.01, ***, *p* < 0.001, ****, *p* < 0.0001). Three biological replicates per dataset were included

The effect of harmaline on bacterial efflux activity was further evaluated using Rhodamine B and ethidium bromide (EtBr) efflux assays. As depicted in Fig. 4A and B, harmaline addition eliminated the efflux of Rhodamine in *K. pneumoniae* T2 [27]. The application of harmaline alone led to a significant increase in fluorescence intensity (Fig. 4C). When used in combination with tigecycline, the fluorescence intensity showed a dose-dependent increase compared to tigecycline alone (Fig. 4D). Collectively, the overall results revealed that harmaline potentiated tigecycline activity by inhibiting the activity of the efflux pump.

**Fig. 5:**
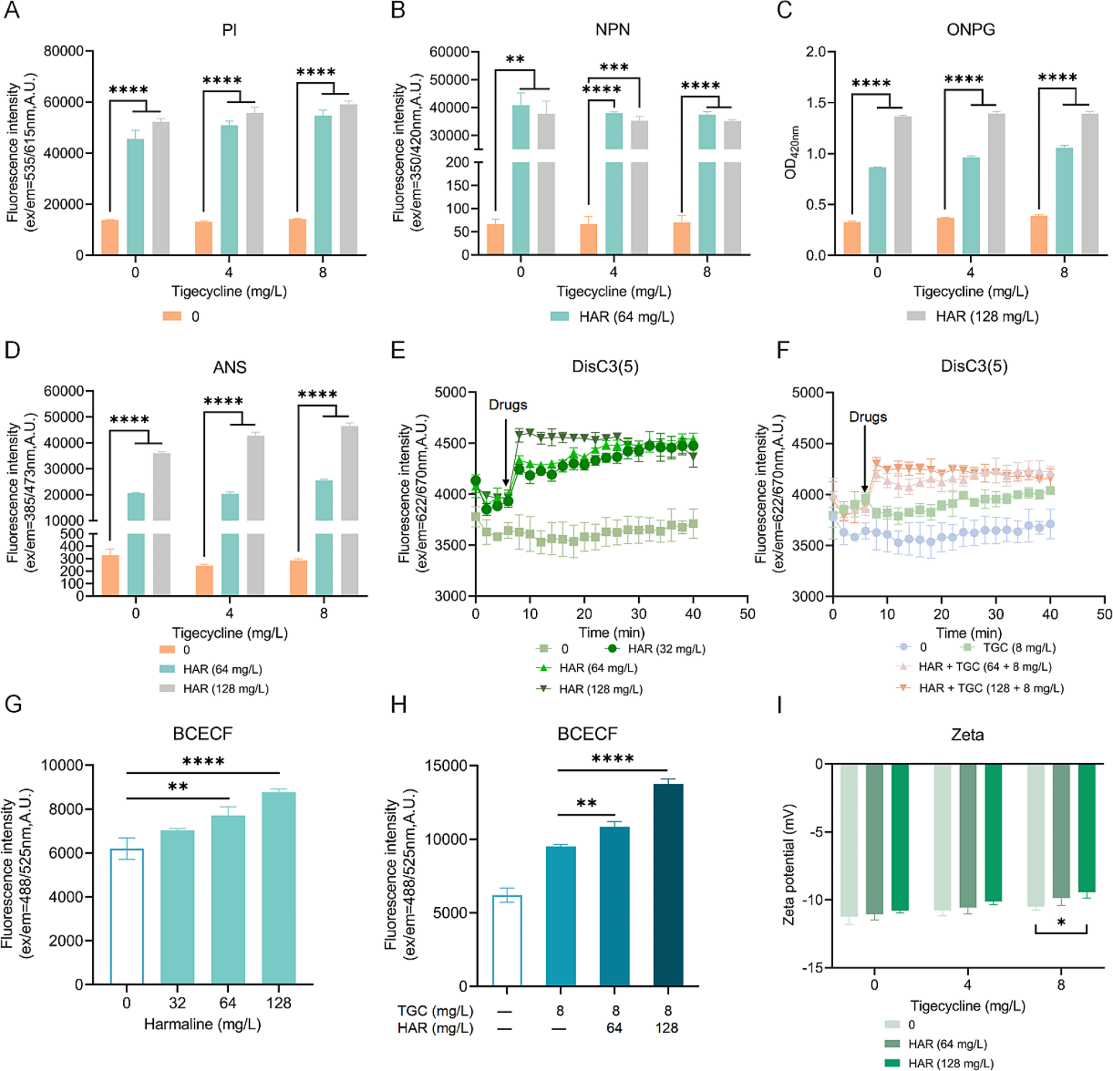
Harmaline disrupts cell membrane permeability and integrity and dissipates the bacterial proton motive force. **A-B** The permeability of the bacterial membranes were determined using PI and NPN, respectively. Then, bacterial membranes destruction with the presence or absence of harmaline and/or tigecycline was determined by β-galactosidase activity **C** and ANS analyses **D**. **E-F** Fluorescence changes in DisC_3_(5) with diverse concentrations of harmaline and/or tigecycline treatment to measure the bacterial transmembrane potential. **G-H** After the treatment of diverse concentrations of harmaline, or in combination with tigecycline, the bacterial ΔPH levels were determined by the pH indicator BCECF-AM. **I** ζ-potential was measured to characterize the changes in bacterial membrane potential (*, *p* < 0.05, **, *p* < 0.01, ***, *p* < 0.001, ****, *p* < 0.0001). Three biological replicates were included

To further elucidate the potential mechanisms underlying the harmaline-mediated inhibition of efflux activity, the membrane permeability of *K. pneumoniae* T2 was further determined using the fluorescence probes propidium iodide (PI) and N-phenyl-1-naphthylamine (NPN). As shown in Fig. 5A and B, membrane permeability was observably increased in the presence of harmaline, as evidenced by the increase in fluorescence intensity. Additionally, the leakage of intracellular β-galactosidase and increased intensity of 8-anilino-1-naphthalenesulfonic acid (ANS) fluorescence further confirmed the disruption of cell membrane integrity (Fig. 5C and D).

Accumulating evidence has shown that efflux pumps are driven by the proton motive force (PMF) [28], which consists of the ∆ψ (membrane potential) and the ∆pH (proton gradient). We hypothesized that harmaline may dissipate bacterial PMF and thereby antagonized the efflux of tigecycline. To test this hypothesis, the fluorescence changes of DisC3(5) and BCECF were monitored. As expected, harmaline treatment dissipated the proton motive force (PMF), as evidenced by the disruption of the ∆ψ (membrane potential; Fig. 5E and F) and the ∆pH (proton gradient; Fig. 5G and H). Additionally, the electronegativity change with the combination of harmaline and tigecycline was significantly higher than tigecycline alone (Fig. 5I). Cumulatively, these results demonstrated that harmaline administration markedly suppressed PMF-driven efflux.

### Mechanistic studies on the interaction of harmaline with TMexCD1-TOprJ1

**Fig. 6:**
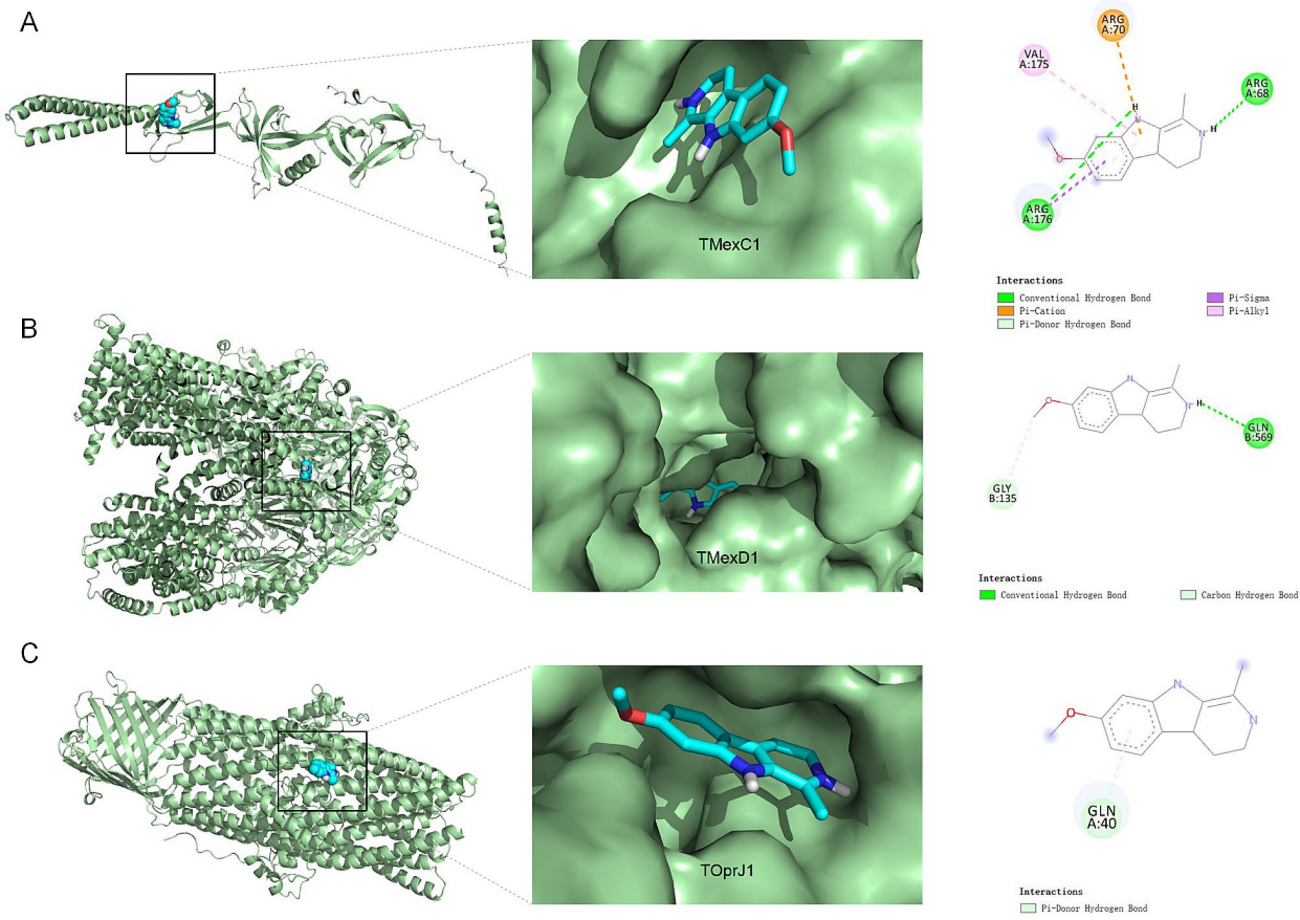
Predicted binding patterns of harmaline to the TMexC1, TMexD1, and TOprJ1 proteins. Binding of harmaline to the TMexC1 **A**, TMexD1 **B,** and TOprJ1 **C** proteins were determined by molecular docking

To further elucidate the potential mechanisms of interaction, molecular docking was employed to identify the binding sites and modes of interaction between harmaline and TMexC1, TMexD1, and TOprJ1. Figure 6A depicted the overall, local, and 2D binding models of harmaline with the TMexC1 protein. The interactions involved hydrogen bonding with ARG176 and ARG68, Pi-Sigma interactions with ARG176, Pi-alkyl interactions with VAL175, and Pi-cation interactions with ARG70. As illustrated in Fig. 6B, harmaline was attached to the active site residues GLY135 and GLN569 in TMexD1 via hydrogen bonding interactions. Additionally, GLN40 residue was involved in hydrogen bonding with TOprJ1, as shown in Fig. 6C. The combined interactions of harmaline with the TMexC1/TMexD1/TOprJ1 active sites highlights the strength of interaction needed for harmaline to interfere with efflux pump activity.

Amino acid site-specific mutations were introduced to validate the binding sites for molecular docking. MIC values against tigecycline were higher in *E. coli* harboring *tmexC1*_(V175I)_, *tmexD1*_(G135V)_, or *toprJ1*_(Q40R)_ than in wide-type strains of *tmexC1*, *tmexD1*, or *toprJ1* (MIC < 1) (Figure S3A, C, and E), which is in line with the previous findings that isolates harboring a portion of *tmexD1*/*toprJ1* or missing *tmexC1* are tigecycline susceptible and *tmexCD1-toprJ1* leading to tigecycline resistance in *K. pneumoniae* [6]. Furthermore, there was no synergistic effect of harmaline and tigecycline in both wide-type strains or the mutations (FIC > 0.5), suggesting that the complete *tmexCD1-toprJ1* gene cluster is needed to mediate tigecycline resistance (Figure S3B, D, and F).

**Fig. 7:**
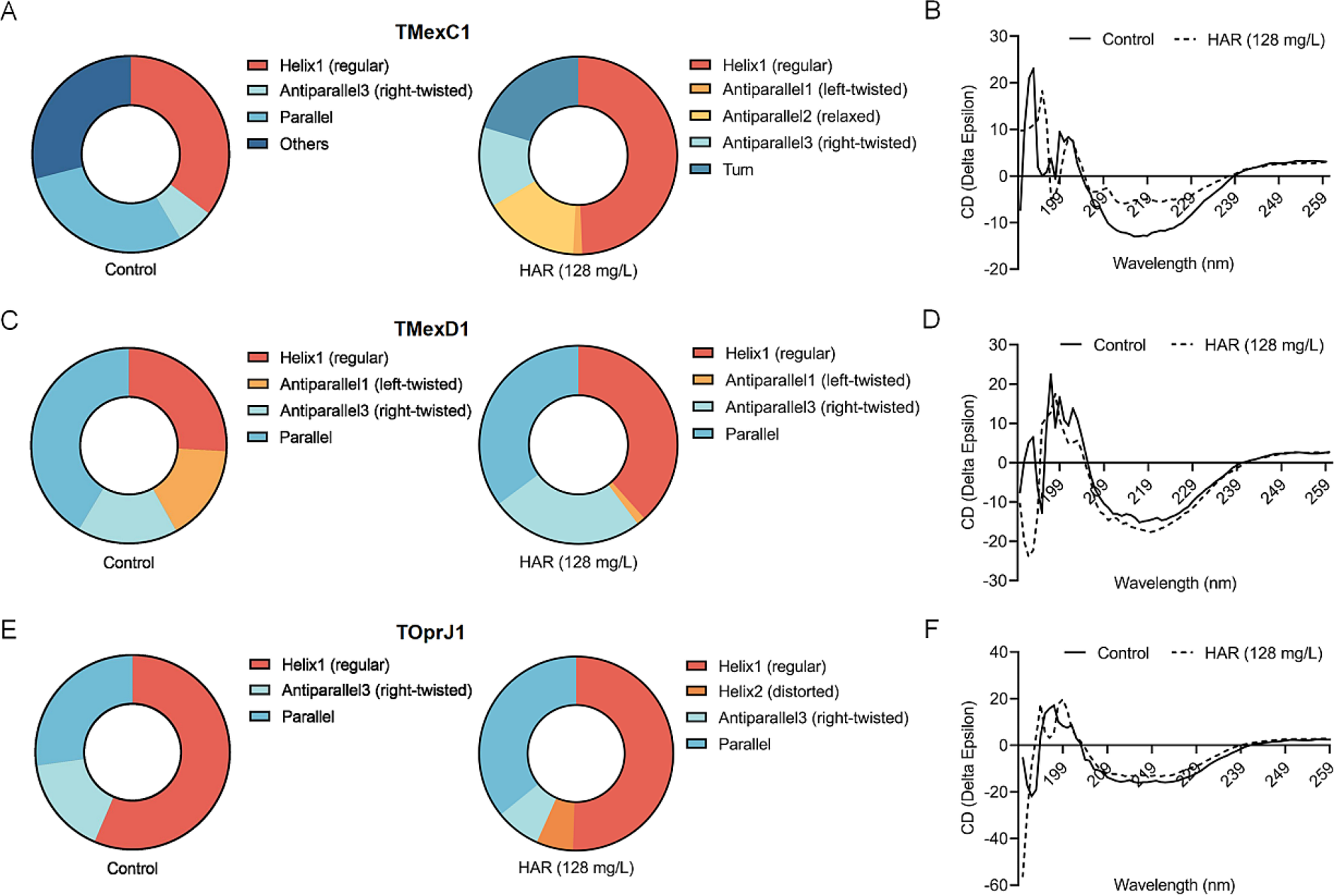
Harmaline altered the secondary structure of the TMexC1, TMexD1, and TOprJ1 proteins. Following induction with or without harmaline at a final concentration of 128 mg/L, the ratio of secondary structure of the TMexC1 **A-B**, TMexD1 **C-D** and TOprJ1 **E-F** proteins were determined

Circular dichroism spectroscopy was used to further test whether harmaline affected the secondary structure of TMexC1/TMexD1/TOprJ1. Significant changes in the structure of TMexC1 (Fig. 7A and B), TMexD1 (Fig. 7C and D) and TOprJ1 (Fig. 7E and F) were observed by harmaline treatment. Concretely, upon exposure to harmaline, TMexC1 and TMexD1 showed an increase in the helix 1 binding, and a decrease in the antiparallel 3 (right-twisted) binding of TOprJ1. Collectively, the interactions of harmaline with TMexC1/TMexD1/TOprJ1 active residues altered the secondary structures.

**Fig. 8:**
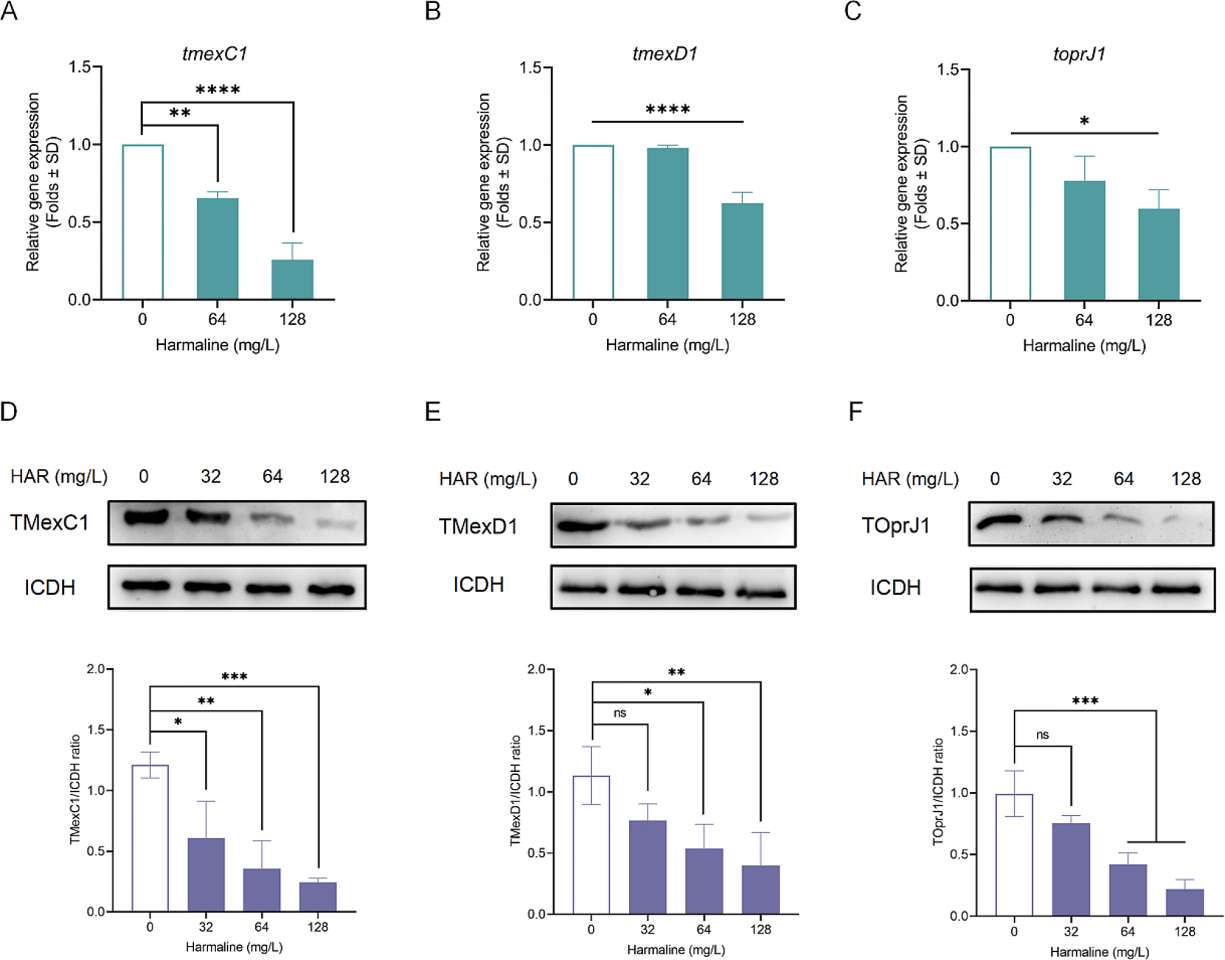
Harmaline inhibits the transcription of *tmexC1*/*tmexD1*/*toprJ1* and expression of TMexC1/TMexD1/TOprJ1. The transcription of *tmexC1***A**, *tmexD1***B**, and *toprJ1***C** were dose-dependently inhibited by harmaline, as determined by RT‒PCR. The expression of TMexC1 **D**, TMexD1 **E,** and TOprJ1 **F** with the presence of harmaline (0, 32, 64, or 128 mg/L) were determined by western blotting analysis (ns, *p* ≥ 0.05, *, *p* < 0.05, **, *p* < 0.01, ***, *p* < 0.001, ****, *p* < 0.0001). Three biological replicates per dataset were included

To further verify whether harmaline directly inhibited the gene transcription of *tmexCD1-toprJ1* and the production of TMexC1/TMexD1/TOprJ1, RT-PCR experiments, and western blotting experiments were performed. As shown in Fig. 8A through C, the transcriptional regulation of *tmexC1*, *tmexD1*, and *toprJ1* were inhibited by harmaline administration in a dose-dependent manner. Congruously, harmaline-mediated decrease of TMexC1/TMexD1/TOprJ1 production was observed in *tmexCD1-toprJ1*-harboring *K. pneumoniae ***(**Fig. 8D **through F**). Overall, these data demonstrated that harmaline administration observably repressed the transcription and expression of TMexC1/TMexD1/TOprJ1.

### Harmaline disrupts redox and metabolic processes in *tmexCD1-toprJ1*-positive *Klebsiella pneumoniae*

**Fig. 9:**
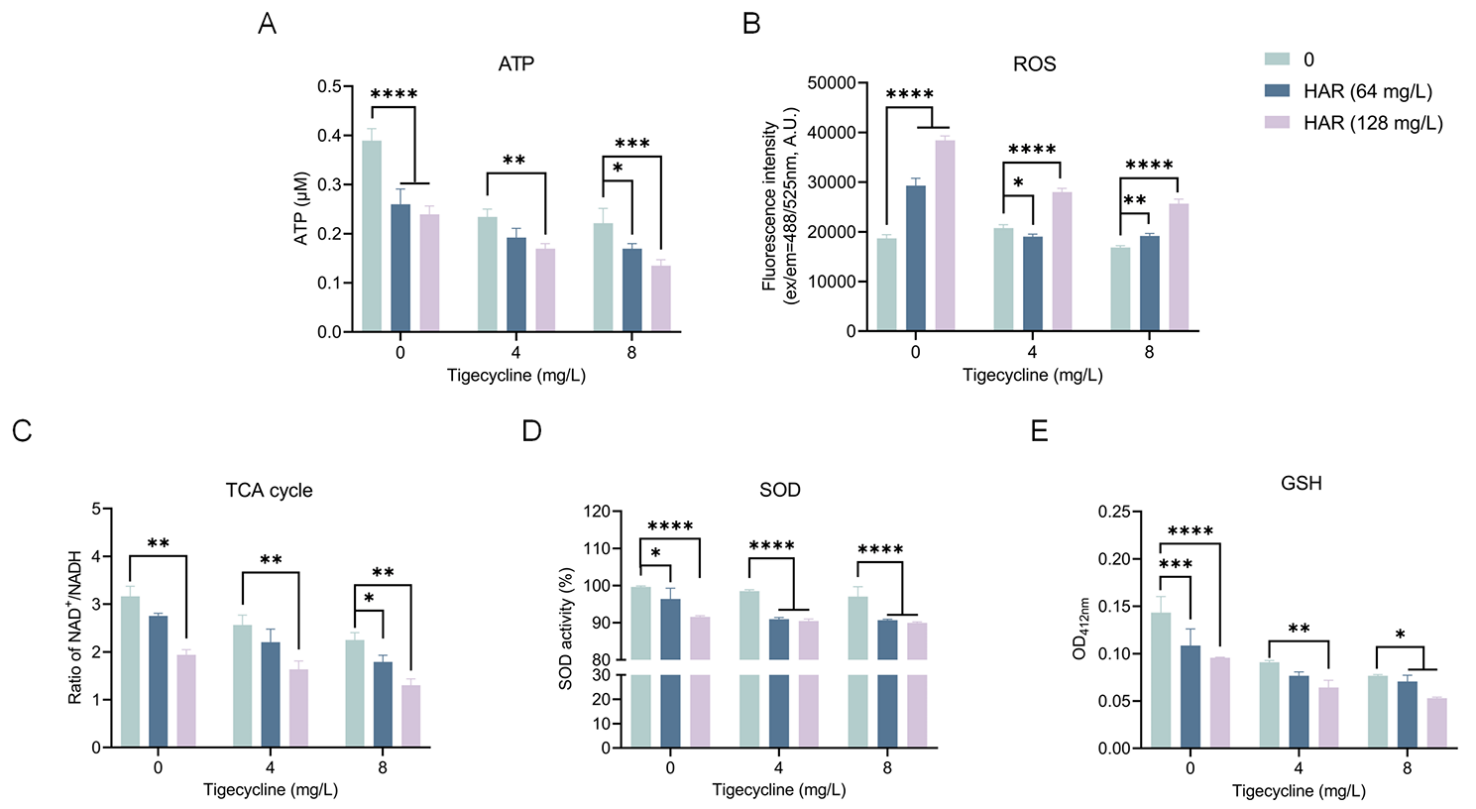
Harmaline disrupts oxidative stress processes in *tmexCD1-toprJ1*-positive bacteria. **A** ATP levels in bacteria after harmaline and/or tigecycline treatment. **B** Changes in bacterial ROS with harmaline alone or combined with tigecycline treatment. **C** NAD^+^/NADH levels in bacteria treated with harmaline, tigecycline or the combination. SOD activity **D** and GSH level **E** treated with harmaline and/or tigecycline. (*, *p* < 0.05, **, *p* < 0.01, ***, *p* < 0.001, ****, *p* < 0.0001). Three biological replicates per dataset were included

Previous studies have confirmed that the PMF is associated with changes in ATP levels and increases in reactive oxygen species (ROS) [29]. Therefore, the levels of ATP and ROS in *K. pneumoniae* T2 were measured. Treatment with harmaline significantly reduced ATP levels in a dose-dependent manner (Fig. 9A). Furthermore, co-incubation with harmaline increased ROS levels compared to individual treatment with tigecycline alone (Fig. 9B). Additionally, compared to tigecycline treatment, harmaline plus tigecycline treatment resulted in a significantly decreased NAD+/NADH, indicating a noteworthy promotion of the TCA cycle (Fig. 9C). SOD activities were further detected to evaluate the oxidation status of *K. pneumoniae* T2 when treated with harmaline or tigecycline. The combinations of harmaline and tigecycline significantly decreased SOD activity compared to tigecycline treatment alone (Fig. 9D). Changes in the level of glutathione (GSH) are also associated with oxidative stress [30]. The GSH-based assay showed that harmaline reduced GSH levels, regardless of the presence of tigecycline **(**Fig. 9E). Thus, these results established that harmaline alters bacterial metabolism, with consequent inhibition of efflux pump function, thereby enhancing the antimicrobial activity of tigecycline.

### Harmaline restores the in vivo activity of tigecycline

Safety has always been a key indicator in evaluating the clinical application of drugs. Firstly, harmaline cytotoxicity was evaluated using two cell lines (Caco-2, J774A.1), which showed no significant cytotoxicity (Figure S4A). Additionally, after oral administration of harmaline at a dose of 200 mg/kg, the mice showed no significant increase in body weight over a period of 14 days, and no adverse effects were observed (Figure S4B). The safety of harmaline in combination with tigecycline was evaluated in cancer cell lines (Caco-2, J774A.1), and the results demonstrated that there was no striking cytotoxicity in harmaline in combination of tigecycline in Caco-2 and J774A.1 cells at the effective concentrations (Figure S4C). These results suggested that harmaline and tigecycline are safe to use at effective concentrations.

**Fig. 10:**
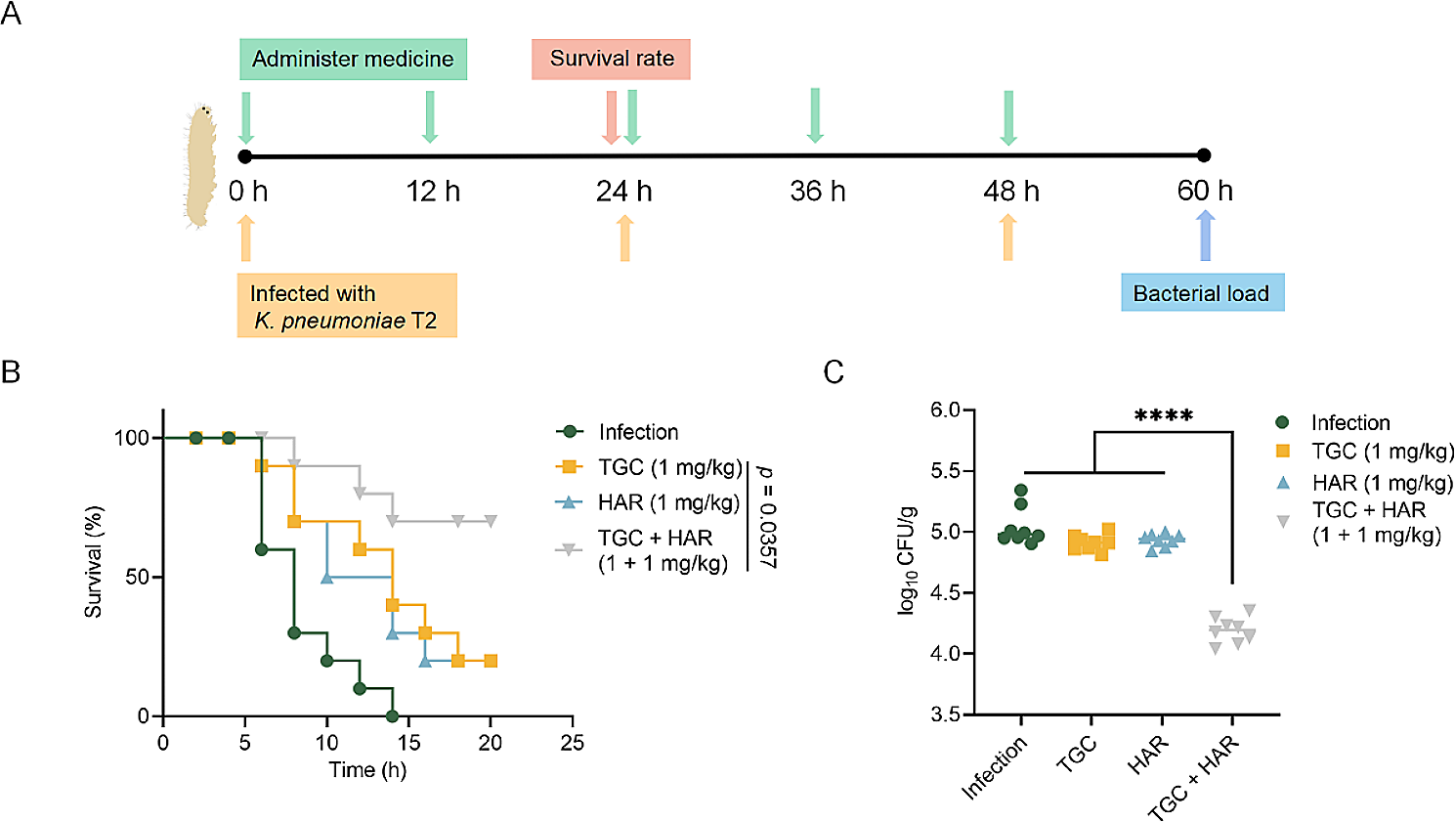
Harmaline effectively improved tigecycline activity in *Galleria mellonella* infection models. **A** The scheme of the survival rate and colonization experiments of *Galleria mellonella*. **B** Survival rates of *Galleria mellonella* infected with *K. pneumoniae* T2 (10^6^ CFUs) (*n* = 10 each group). *p* value was determined by the log-rank (Mantel-Cox) test. **C** Colonization of *Galleria mellonella* infected by *K. pneumoniae* T2 (2 × 10^5^ CFUs) (*n* = 8 each group) and then treated with PBS, tigecycline (1 mg/kg), harmaline (1 mg/kg), or a combination of harmaline plus tigecycline (1 + 1 mg/kg) (****, *p* < 0.0001)

Considering the combination of harmaline and tigecycline displayed outstanding synergistic activity against *tmexCD1-toprJ1-*positive strains in vitro, we further evaluated the synergistic effects in vivo. First, to test this, the method shown in Fig. 10A was used to assess the effect of tigecycline or harmaline monotherapy or tigecycline/harmaline combination therapy in *Galleria mellonella* models. In the survival study, all *Galleria mellonella* died within 20 h in the infected group. Notably, the combination therapy group dramatically improved survival (70%), compared to the harmaline/tigecycline-treated group (20%) (Fig. 10B). Consistently, in the colony forming assay, compared with harmaline/tigecycline monotherapy, the combination therapy group exhibited a remarkable reduction in bacterial load (Fig. 10C).

**Fig. 11:**
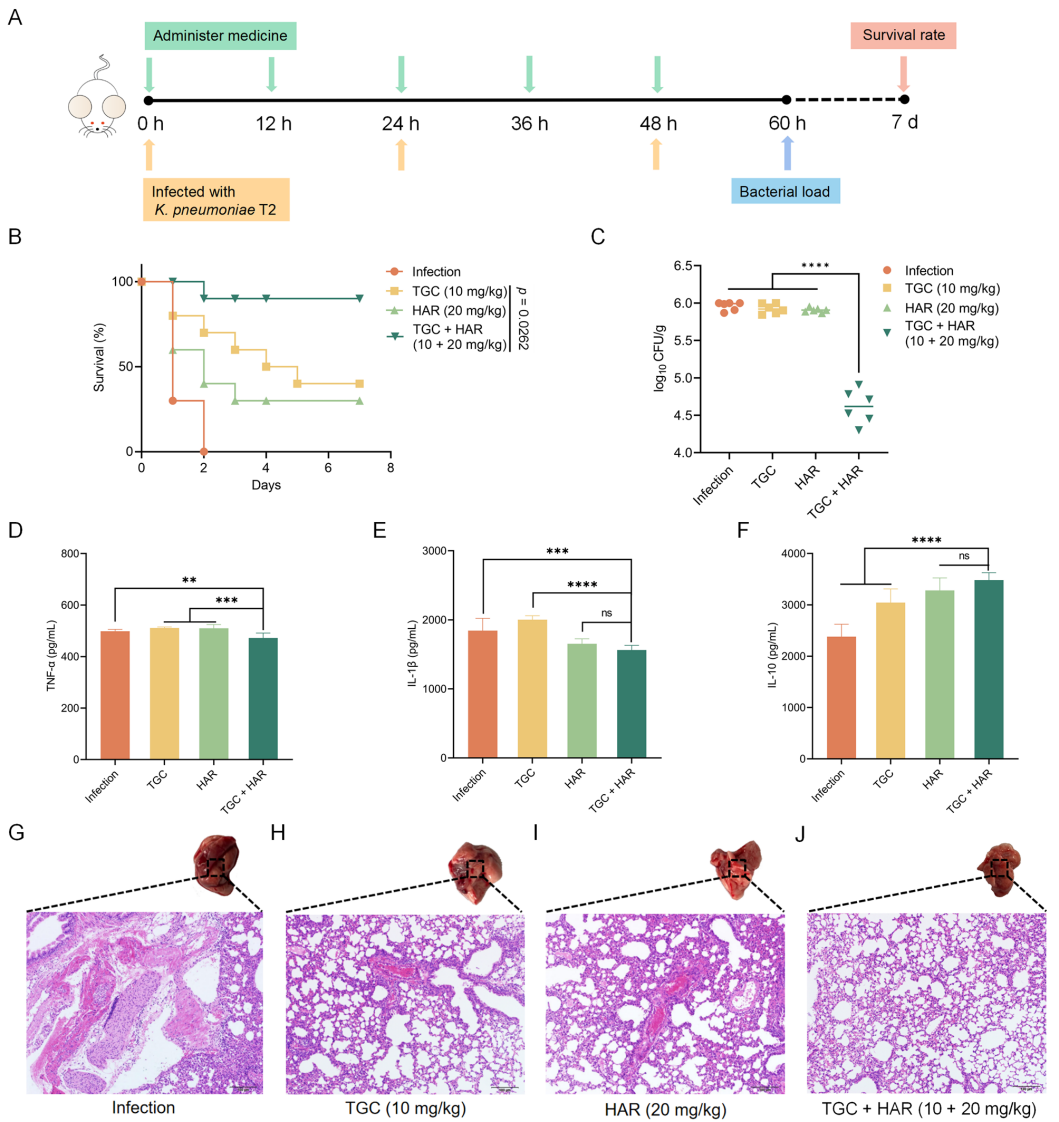
Harmaline effectively improved tigecycline activity in *K. pneumonia* T2 infected mice. **A** Protocols for mice survival and colonization experiments. **B** Survival rates of mice infected with *K. pneumoniae* T2 (1.6 × 10^10^ CFUs) (*n* = 10 each group) and then treated with PBS, tigecycline (10 mg/kg), harmaline (20 mg/kg), or their combination. *p* value was determined by the log-rank (Mantel-Cox) test. **C** Colonization of mice infected with *K. pneumoniae* T2 (10^7^ CFUs) (*n* = 6 each group) and then treated with PBS, tigecycline (10 mg/kg), harmaline (20 mg/kg), or a combination of harmaline plus tigecycline (20 + 10 mg/kg) (****, *p* < 0.0001). **D-F** The production of inflammatory cytokines in lungs were determined using enzyme-linked immunosorbent assay (ELISA). Three biological replicates per dataset were included. (ns, *p* ≥ 0.5, **, *p* < 0.01, ***, *p* < 0.001, ****, *p* < 0.0001). **G-J** The pathological injury of lungs were evaluated by gross observation and pathologic H&E staining

Apart from this, *K. pneumoniae* T2-infected mouse models were employed to explore the in vivo synergistic effects of harmaline plus tigecycline against *tmexCD1-toprJ1*-positive *K. pneumoniae* (Fig. 11A). In the survival rate experiment, after 7 days, the survival rate increased to 90% in the combination group compared to the control group. Interestingly, the harmaline and tigecycline monotherapy groups also showed effective protection against infected group, with survival rates of 30% and 40%, respectively (Fig. 11B). Furthermore, in the colony forming assay, the combination of tigecycline and harmaline demonstrated more effective CFU reduction compared to tigecycline/harmaline monotherapy (Fig. 11C). In addition, pro-inflammatory factor TNF-α/IL-1β and anti-inflammatory cytokine IL-10 associated with cell injury and tissue repair [31, 32]. To evaluate the effect of harmaline on the inflammatory response in mice, TNF-α, IL-1β, and IL-10 levels were monitored. Compared with tigecycline/harmaline monotherapy, the combination therapy significantly suppressed TNF-α and IL-1β levels and promoted IL-10 production, suggesting that harmaline is also involved in tigecycline modulation of the immune response to infection (Fig. 11D through F). Correspondingly, the gross observations of intact lungs or histopathology of HE-stained sections were used to evaluate treatment efficacy. In an infected and untreated lung, pulmonary congestion and edema were visible. Hemocyte aggregation, diffuse cellular damage, and neutrophil infiltration were further observed in HE sections. Compared to harmaline/tigecycline treatment alone, the combination therapy resulted in significant improvement of lung lesions and marked abatement of inflammatory cell infiltrates (Fig. 11G through J). In conclusion, these in vivo effective results strongly demonstrated the potential of harmaline in combination with tigecycline in combating infectious diseases caused by *tmexCD1-toprJ1*-positive bacteria.

## Discussion and conclusion

The overuse of antibiotics and the lack of innovation in developing new antimicrobial drugs have led to the emergence of drug-resistant pathogens, which pose a considerable threat to modern medicine. *K. pneumoniae* is one of the leading drug-resistant bacteria worldwide because of its ability to acquire drug resistance genes and its speed of evolution. Tigecycline has been considered the last line of defense for treating intricate infections caused by multidrug-resistant gram-negative pathogens [33]. However, the appearance and rapid global spread of *tmexCD1-toprJ1* plasmid-mediated tigecycline resistance have greatly reduced the effectiveness of tigecycline [34].

This research aimed to screen nonantibiotic adjuvants that could inhibit the efflux pump mediated by *tmexCD1-toprJ1*, thereby restoring the efficacy of tigecycline against drug-resistant bacteria. Based on our previous work, we used the checkerboard method to screen target compounds from our natural drug libraries. Harmaline was found to synergistically enhance tigecycline activity against *tmexCD1-toprJ1-*carrying bacteria. Therefore, we chose harmaline for further study. Through antimicrobial susceptibility tests, time-dependent killing experiments, post antibiotic effect tests and combination disc tests, we found that harmaline showed significant bactericidal activity when used alone or in combination with tigecycline, and its bactericidal effect varied with time, which highlights the efficacy of the drug combination. Reliable antibiotic adjuvants not only enhance antibiotic activity but also hinder the development of resistance. Resistance evolution analysis showed that compared with the tigecycline-treated group (8-fold increase in the MIC), there was no significant change in the MIC value of the group treated with tigecycline and harmaline, indicating that harmaline is not easy to induce bacterial resistance against tigecycline. The results of the mutant prevention concentration test demonstrated that MPC and MPC/MIC decreased with the increase of harmaline concentration, which further proved that the mutation selection window of tigecycline became narrower under the effect of harmaline. These results indicated that harmaline enhanced the antibacterial activity of tigecycline and prevented the evolution of tigecycline resistance as well.

Subsequently, we used *tmexCD1-toprJ1*-positive *K. pneumoniae* T2 as a test strain to investigate the mechanism by which harmaline enhances the antibacterial activity of tigecycline. Mechanistically, to test the function of the efflux pump, we performed Rhodamine and EtBr test experiments, and as expected, the function of the bacterial efflux pump was disrupted. Harmaline was found to disrupt bacterial membranes by inner/outer membrane permeability test, extracellular β-galactosidase measurement, membrane fluidity assay. By detecting the level of fluorescence in DisC3(5) and BCECF experiments, it was demonstrated that the disruption of the bacterial membrane affected the bacterial proton motive force (PMF), which is essential for efflux pump function. Molecular docking assays predicted the binding sites of harmaline to TMexC1/TMexD1/TOprJ1 interacting subunits, and the amino acid site-specific mutation confirmed the prediction. By circular dichroism spectra analysis, we found that harmaline altered the secondary structure of the TMexC1, TMexD1, and TOprJ1 proteins. In addition, we determined the transcription levels of *tmexC1*, *tmexD1*, and *toprJ1* by RT‒PCR and expression levels of TMexC1/TMexD1/TOprJ1 by western blotting experiments under treatment with different concentrations of harmaline, which showed a harmaline dose-dependent decrease. In view of the important role of bacterial metabolism in the efficacy of antibiotics, we further explored whether harmaline affected bacterial metabolic homeostasis. Furthermore, harmaline diminished bacterial ATP levels, leading to suppressed efflux pump function, facilitated ROS accumulation, and increased bactericidal activity of tigecycline. Harmaline also affected the bacterial oxidative environment and metabolic levels by altering the NAD^+^/NADH, SOD, and GSH levels. It is concluded that, harmaline is able to alter the metabolic homeostasis of bacteria and thus enhance the antibacterial activity of tigecycline.

Safety assessments through cell cytotoxicity and mouse toxicity experiments gave satisfactory results before we evaluated the in vivo effects of harmaline. The synergistic activity of harmaline and tigecycline was validated in both the *Galleria mellonella* infection models and the mouse infection models. In terms of in vivo effect evaluation, co-treatment of harmaline and tigecycline significantly increased the survival rate and decreased the bacterial load of the *Galleria mellonella* and mice. The results of cellular inflammation level assay showed that TNF-α and IL-1β levels were significantly decreased and IL-10 levels were dramatically increased in the combination treatment group compared with tigecycline/harmaline alone, suggesting that harmaline was involved in the regulation of inflammation in the bacterially-infected hosts. The results of H&E sections demonstrated that the combination treatment significantly ameliorated the lung lesions of mice, and markedly attenuated the inflammatory cell infiltration. The above results suggest that harmaline can reverse the high level of tigecycline resistance mediated by *tmexCD1-toprJ1*in vivo, and the combined effect has good therapeutic activity.

In conclusion, harmaline has shown efficacy as an adjuvant to tigecycline and can be used in combinations to treat infections caused by *tmexCD1-toprJ1-*positive pathogens. These findings offer a new perspective for the discovery of tigecycline adjuvants. In this work, solvents should be further improved or different dosage forms should be made to increase the solubility of harmaline, and the drug safety window should be expanded to increase the maximum safe dose for application in clinical studies. In addition, additional research is urgently needed to study the structure of harmaline for structure-activity relationship optimization of the killing of MDR bacterial pathogens.

### Electronic supplementary material

Below is the link to the electronic supplementary material.

Supplementary Material 1

Supplementary Material 2

## Data Availability

The data would be available from the corresponding author upon reasonable request.
