# Supplementary material for: Repurposing harmaline as a novel approach to reverse tmexCD1-toprJ1-mediated tigecycline resistance against klebsiella pneumoniae infections
Source: Microb Cell Fact. 2024 May 24;23:152. doi: 10.1186/s12934-024-02410-4 (PMC11127330; doi:10.1186/s12934-024-02410-4)
Supplement: Supplementary file 1 — Supplementary Material 1 [file 12934_2024_2410_MOESM1_ESM.docx]

**Supplementary Table 1** The tested bacterial hosts carrying *tmexCD1-toprJ1*-positive plasmids and their transconjugants or transformants in this study.

| **Species** | **Resistance gene** | **Geographical positions** | **Source/Description** |
| --- | --- | --- | --- |
| *K. pneumoniae* T1 | *tmexC1-toprJ1* | - | Chicken |
| *K. pneumoniae* T2 | *tmexC1-toprJ1* | - | Chicken |
| *K. pneumoniae* T3 | *tmexC1-toprJ1* | - | Chicken |
| *K. pneumoniae* T4 | *tmexC1-toprJ1* | - | Chicken |
| *E. coli* DH5α-pET28a-*tmexC1* | *tmexC1* | - | Transformants |
| *E. coli* DH5α-pGEX-6P-1-*tmexD1* | *tmexD1* | - | Transformants |
| *E. coli* DH5α-pET28a-*toprJ1* | *toprJ1* | - | Transformants |
| *E. coli* BL21-pET28a-*tmexC1* | *tmexC1* | - | Transformants |
| *E. coli* BL21-pGEX-6P-1-*tmexD1* | *tmexD1* | - | Transformants |
| *E. coli* BL21-pET28a-*toprJ1* | *toprJ1* | - | Transformants |

**Supplementary Table 2** Sequence of primers used for point mutation.

| **Gene** | **Primer** | **Sequence (5´-3´)** |
| --- | --- | --- |
| *tmexC1***_(V175I)_** | Forward | GTTATGCCGAGATTCGCGCCCCC |
|  | Reverse | GGGGGCGCGAATCTCGGCATAAC |
| *tmexD1***_(G135V)_** | Forward | GCAGGCCACCGCCGTCTTTCTGCTGATCT |
|  | Reverse | AGATCAGCAGAAAGACGGCGGTGGCCTGC |
| *toprJ1***_(Q40R)_** | Forward | GTCGCACTGGCGTGCCGCCGACGC |
|  | Reverse | GCGTCGGCGGCACGCCAGTGCGAC |

**Supplementary Table 3** Sequence of primers used for protein purification and site-directed mutagenesis.

| **Gene** | **Primer** | **Sequence (5´-3´)** |
| --- | --- | --- |
| *tmexC1* | Forward | cgc**ggatcc**atggatcactttttccgtga |
|  | Reverse | ccg**ctcgag**ttactgggcttgggactgtg |
| *tmexD1* | Forward | cgc**ggatcc**tccgacgccccaattttgcc |
|  | Reverse | ccg**ctcgag**ttactccgctttatgcaggg |
| *toprJ1* | Forward | cgc**ggatcc**atgacctctcacttcatgct |
|  | Reverse | ccg**ctcgag**tcactcagcgccgatcccgg |

Restriction enzyme sites are underlined and bold.

**Supplementary Table 4** Sequence of primers used for RT-PCR.

| **Gene** | **Primer** | **Sequence (5´-3´)** |
| --- | --- | --- |
| *tmexC1* | Forward | atggatcactttttccgtga |
|  | Reverse | ttactgggcttgggactgtg |
| *tmexD1* | Forward | tccgacgccccaattttgcc |
|  | Reverse | ttactccgctttatgcaggg |
| *toprJ1* | Forward | atgacctctcacttcatgct |
|  | Reverse | tcactcagcgccgatcccgg |
