# Supplementary figures and images for: Repurposing harmaline as a novel approach to reverse tmexCD1-toprJ1-mediated tigecycline resistance against klebsiella pneumoniae infections
Source: Microb Cell Fact. 2024 May 24;23:152. doi: 10.1186/s12934-024-02410-4 (PMC11127330; doi:10.1186/s12934-024-02410-4)

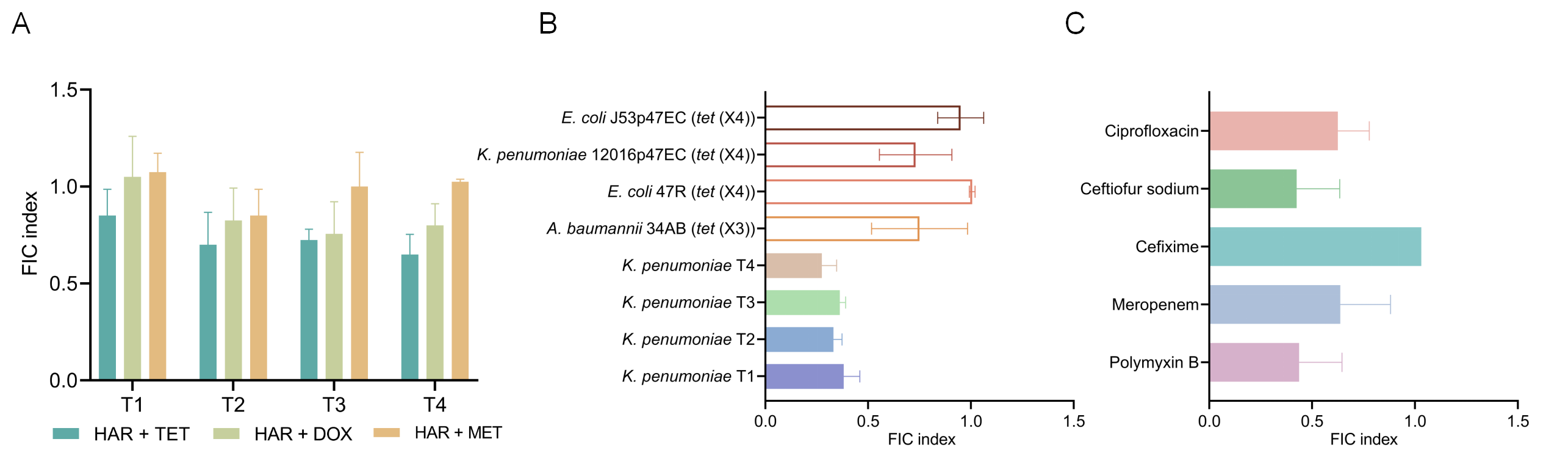

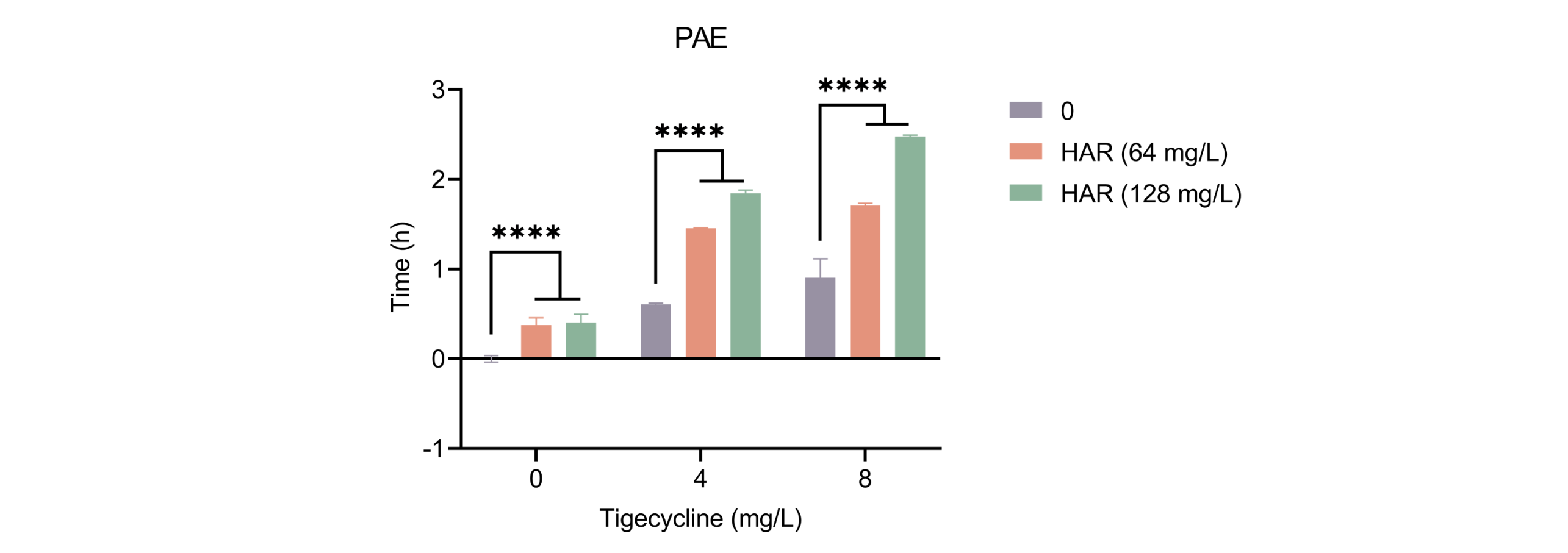

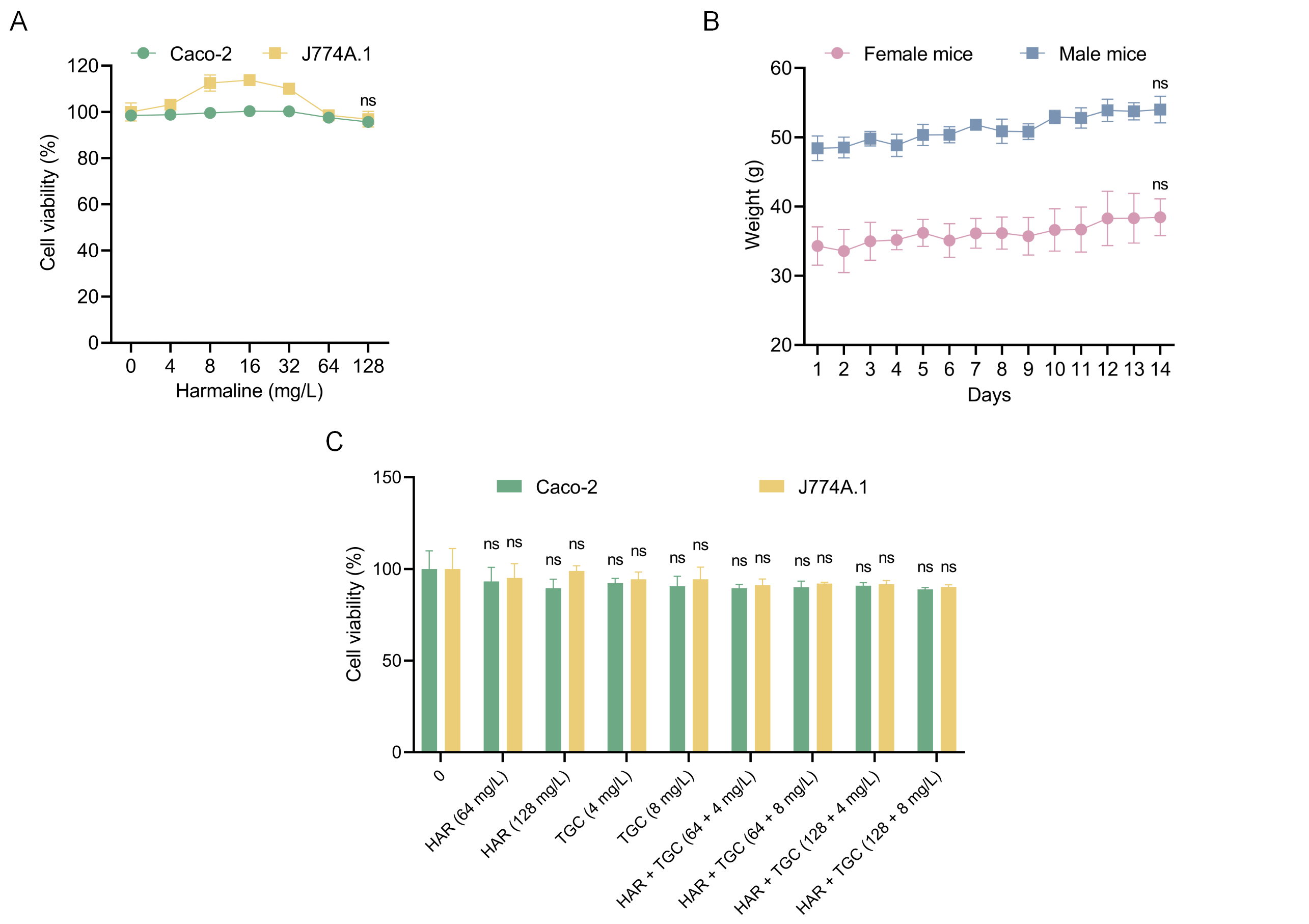

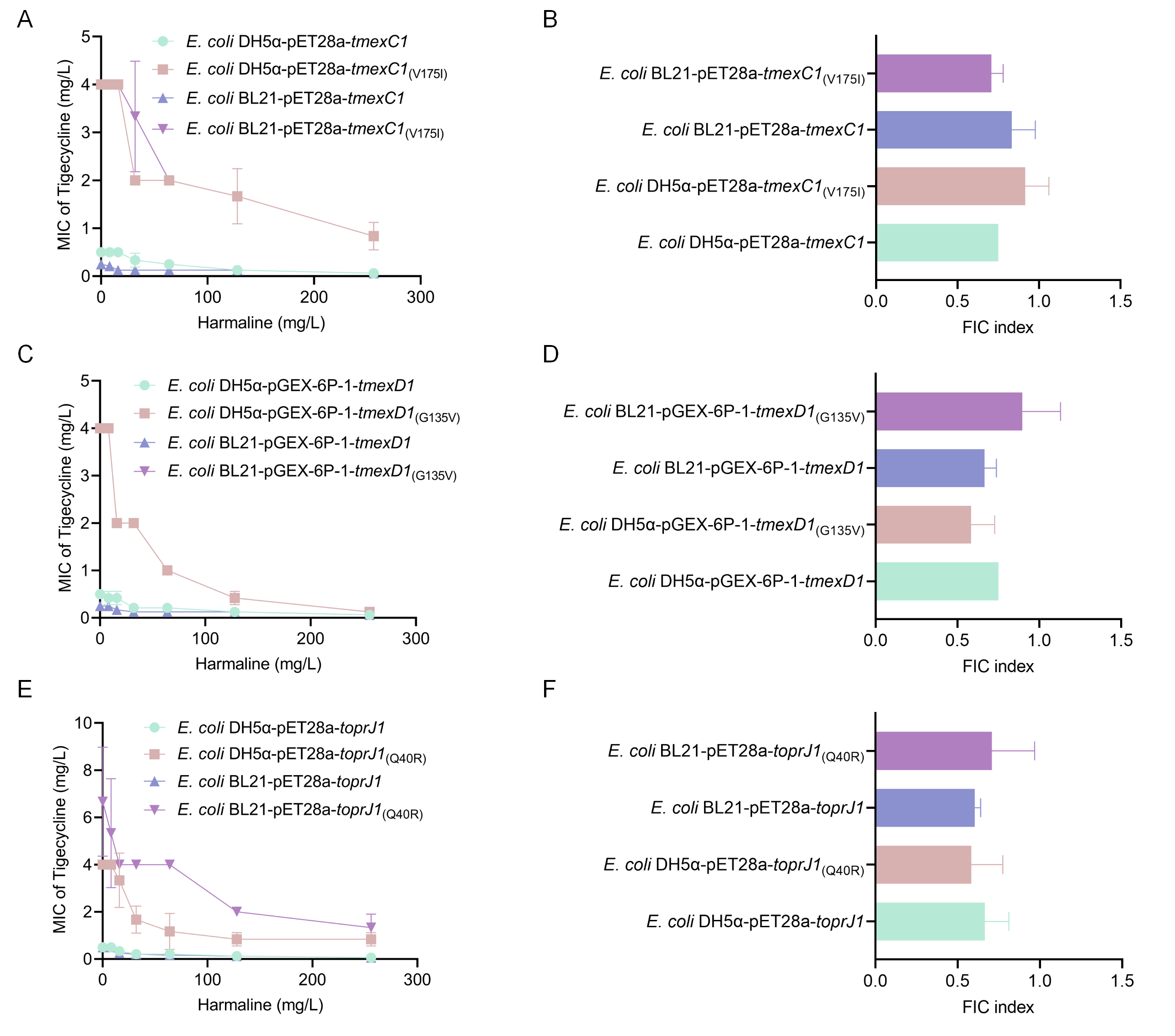

Supplement: Supplementary file 2 — Supplementary Material 2 [file 12934_2024_2410_MOESM2_ESM.docx]
